# Alternative ribosome expression alone is insufficient to drive antibiotic tolerance in *Mycobacterium bovis* BCG

**DOI:** 10.3389/fmicb.2026.1882584

**Published:** 2026-08-24

**Authors:** Rachel E. Butler, James Barber, George Mayson, Gillian Wallis, Graham R. Stewart

**Affiliations:** 1 School of Biosciences, Faculty of Health and Medical Sciences, University of Surrey, Guildford, United Kingdom; 2 Bioimaging and Flow Cytometry Core Facility, School of Biosciences, Faculty of Health and Medical Sciences, University of Surrey, Guildford, United Kingdom

**Keywords:** BCG, mycobacteria, *Mycobacterium tuberculosis*, ribosomal proteins, tolerance, zinc

## Abstract

**Introduction:**

*Mycobacterium tuberculosis* and other members of the *M. tuberculosis*-complex are obligate pathogens of mammals. Their unique life and transmission cycle finds them in micro-environments with extremes in concentration of zinc. In the macrophage, zinc is pumped into the phagosome by the host cell causing zinc poisoning. However once *M. tuberculosis* exits from the phagosome and macrophage, the extracellular milieu and caseum of the tubercle lesion are zinc restricted due to zinc sequestration by the neutrophil protein calprotectin. To counter low zinc levels*, M. tuberculosis* expresses genes that are members of the “Zur” regulon. These include zinc uptake transporters, and five “alternative” ribosome paralogues that are zinc-independent. Under zinc replete conditions, the Zur protein is bound to zinc and acts as a transcriptional repressor, binding to conserved DNA sites in promoter regions of the Zur regulon. During zinc limiting conditions, the apo form of Zur dissociates from promoter regions allowing transcription of the Zur regulon including alternative ribosome proteins. The expression of alternative ribosomes by mycobacteria is of considerable interest due to the association of expression with antibiotic tolerance.

**Methods:**

In this study, we have utilised a fluorescent reporter construct and flow cytometry to evaluate alternative ribosome expression in *M. bovis* BCG under a range of culture conditions and laboratory media.

**Results:**

Our data show two general scenarios under which alternative ribosome expression is induced – under zinc restriction and in extended stationary phase under certain culture conditions. Zinc-restricted bacilli were not found to be more tolerant to antibiotic treatment than those grown in zinc-replete media. For bacilli in extended stationary phase, alternative ribosome expression did not consistently correlate with tolerance to streptomycin or rifampicin.

**Discussion:**

Our data suggest that rather than causing antibiotic tolerance, expression of alternative ribosomes can occur concurrently with other physiological changes that result in antibiotic tolerance.

## 1 Introduction

*Mycobacterium tuberculosis* and other members of the *M. tuberculosis*-complex are obligate pathogens of mammals. Their unique life and transmission cycle finds them in micro-environments with extremes in concentration of zinc. When *M. tuberculosis* aerosol is inhaled and the bacilli are phagocytosed by pulmonary macrophages, zinc is redistributed into the mycobacterium-containing phagosome by the host cell to concentrations up to 459 μM (Wagner et al., 2005). This causes zinc poisoning to the bacteria and restricts infection (Botella et al., 2011). However, once *M. tuberculosis* exit from the phagosome and macrophage, the extracellular milieu and caseum of the granulomatous tubercle lesion are zinc-restricted, as zinc is sequestered by the protein calprotectin that is secreted by neutrophils (Corbin et al., 2008). Zinc is an essential micronutrient as it is a co-factor for enzymes and proteins undertaking diverse roles in bacteria, such as DNA synthesis, DNA transcription to RNA and protein translation, and as such zinc restriction inhibits bacterial growth. Poisoning and restriction of zinc are examples of “nutritional immunity” used to control pathogens (Hood and Skaar, 2012). Furthermore, variation in zinc concentrations that *M. tuberculosis* are exposed to has been postulated to drive phenotypic heterogeneity within the bacterial population.

To counter low zinc levels, *M. tuberculosis* expresses genes that are members of the Zur regulon (Maciag et al., 2007). These include zinc uptake transporters such as that which is associated with the ESX-3 secretion system, and five “alternative” ribosomal protein paralogues (Prisic et al., 2015; Dow and Prisic, 2018; Dow et al., 2021; Dow et al., 2022). Whereas “primary” ribosomes contain proteins with the zinc-dependent CXXC motif and require zinc for their function, alternative ribosomes lack this motif (C-) and do not require zinc for functionality. In *M. tuberculosis*, the alternative ribosome proteins RpmB2 (50S L28), RpmG1 (50S L33), RpsN2 (30S S14) and RpsR2 (30S S18) are expressed together from a single operon (Rv2055c-Rv2058c), whereas the gene encoding RpmB1 (50S L28) is at a different genomic location (Rv0105c) (Kapopoulou et al., 2011). Under zinc replete conditions, Zur protein binds to zinc and acts as a transcriptional repressor at conserved DNA binding motifs. During zinc limiting conditions, the apo form of Zur dissociates from the Zur binding site allowing transcription of the Zur regulon (Lucarelli et al., 2007; Maciag et al., 2007).

The alternative ribosome of *M. tuberculosis* bacilli is of considerable interest due to the association of its expression with antibiotic tolerant phenotypes (Boldrin et al., 2020). Clinical treatment of drug sensitive tuberculosis typically requires 6 months of therapy with rifampicin, isoniazid, ethambutol and pyrazinamide. This is due to a subpopulation of treatment refractive “persister” bacilli that exhibit slower killing kinetics in response to antibiotics due to phenotypic rather than genetic changes. Keren et al. (2011) performed transcriptome analysis on antibiotic persister bacilli, and observed that the majority of genes encoding ribosomal proteins were downregulated with the exception of the zinc independent Zur-regulated alternative ribosome paralogues *rpmB2-rpmG1-rpsN2-rpsR2*. Bacilli in the sputum are thought to be a source of slow growing persister bacilli, and similarly have a transcriptional signature of zinc starvation and alternative ribosome expression (Garton et al., 2008; Lai et al., 2021). Tolerance to antibiotics has been shown to occur during slow growth, and alternative ribosome expression is required for slow growth in a chemostat model (Beste et al., 2007). Furthermore, alternative ribosome expression is induced during stationary phase in some experimental data sets (Voskuil et al., 2004; Butler et al., 2025).

Li et al. (2018) directly investigated the effects of aminoglycoside antibiotics kanamycin and streptomycin on alternative ribosomes in zinc starved *M. smegmatis*. Their data suggested that under mild zinc starvation, alternative ribosomal paralogues are expressed and assemble into alternative ribosomes in mycobacteria. Upon further zinc starvation, the ribosomal hibernation factor “Mycobacterial Protein Y” (MPY) is exclusively recruited to alternative ribosomes by the MPY-recruitment factor “MRF.” MPY binding occludes the binding of kanamycin and streptomycin to the RNA-loading site of the ribosome, conferring antibiotic tolerance of the bacilli to these antibiotics, and resulting in a “hibernating” ribosome that does not translate RNA (Li et al., 2018, 2019, 2021; Li Y. et al., 2020). However, this finding was not without controversy as other groups have demonstrated that MPY does not exclusively bind to ribosomes during zinc starvation, and that zinc-starved alternative ribosomes maintain their translational abilities, albeit with an altered translatome (Chen et al., 2020). Tolerance to aminoglycosides under zinc starvation has not been demonstrated within *M. tuberculosis*-complex organisms.

In this study, we have created a fluorescent reporter construct to measure the activity of the *rpmB2-rpmG1-rpsN2-rpsR2* alternative ribosome expression under a range of culture conditions in the *M. tuberculosis* model organism *M. bovis* BCG. Our data show two scenarios under which the *rpmB2* promoter is active—under zinc restriction and in extended stationary phase in some but not all culture environments. Bacilli from these conditions were monitored for expression of the alternative ribosome alongside testing for antibiotic tolerance to rifampicin and streptomycin to bring clarity to the question of whether alternative ribosome expression is directly linked to antibiotic tolerance in a member of the *M. tuberculosis-*complex.

## 2 Materials and methods

### 2.1 Construction of RpmB2-reporter constructs

**Figure 1:**
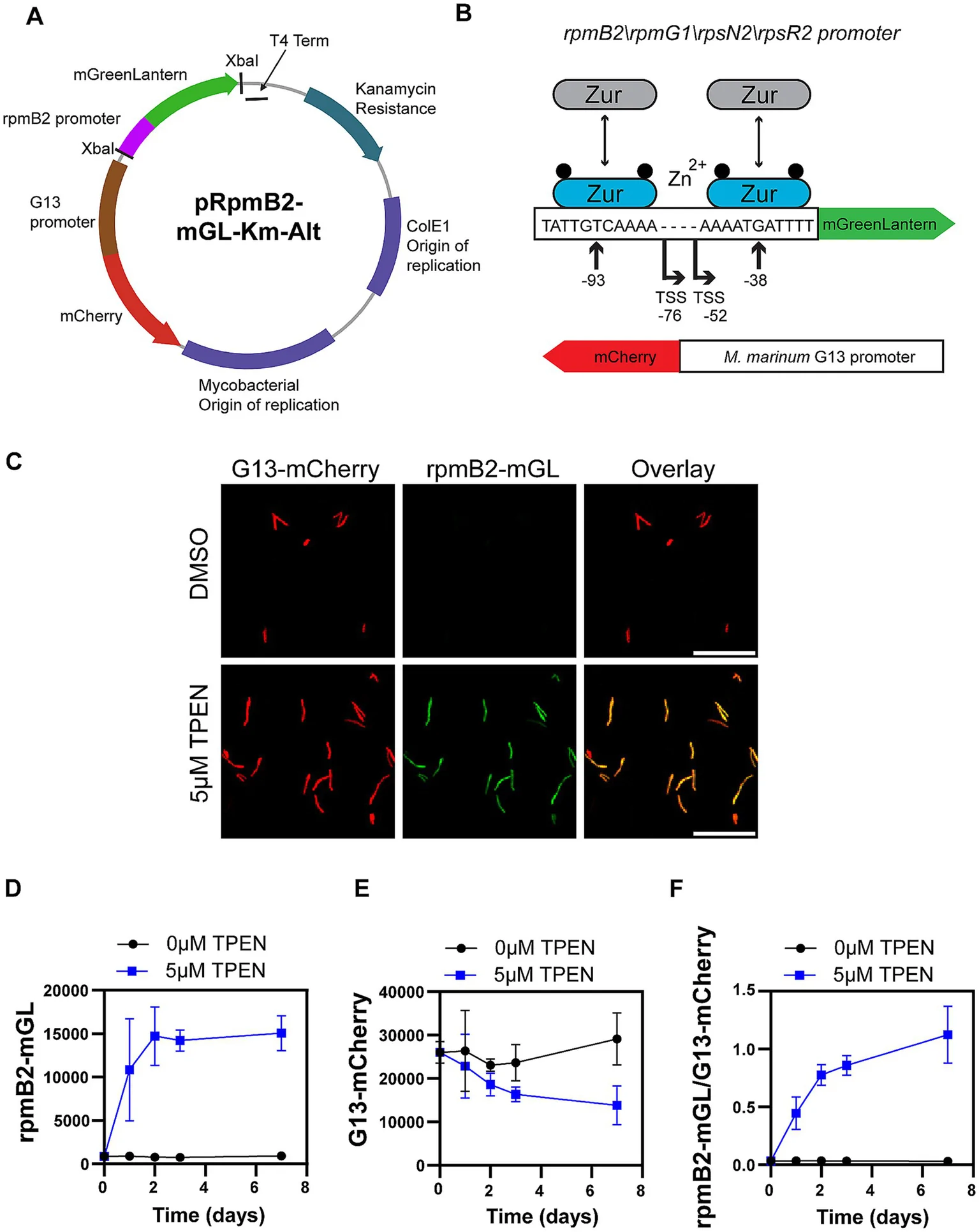
Construction of an alternative ribosome *rpmB2*-mGreenLantern reporter construct. **(A)** Plasmid map of pRpmB2-mGL-Km-Alt alternative ribosome reporter construct. **(B)** Promoter structure of the alternative ribosome reporter construct. Transcriptional start sites (TSS) are from (Cheah et al., 2024). **(C)** *M. bovis BCG* bacilli carrying the reporter construct (BCG pRpmB2-mGL-Km-Alt) were treated with 5 μM TPEN to chelate zinc for 96 h, and analysed by confocal microscopy. Scale bar in white is 20 μm. **(D–F)** *M. bovis BCG* bacilli carrying the reporter construct were treated with 5 μM TPEN to chelate zinc (or DMSO carrier control) for 7 days and analysed by flow cytometry. The median fluorescent intensity of *rpmB2*-mGreenLantern (rpmB2-mGL) **(D)**, G13-mCherry **(E)**, and the ratio of these values **(F)** is plotted. Data are the mean of *N* = 3 independent experiments +/− SD. Created in BioRender. Butler, R. (2026) https://BioRender.com/ifpfast.

The aminoglycoside phosphotransferase gene from pUC4K was cloned into the episomal *Escherichia coli*/mycobacterium shuttle plasmid pSMT176 (Stewart et al., 2004) on a Pst1 restriction fragment to make a kanamycin selectable shuttle plasmid, pG1A. The pG13-mCherry expression cassette was PCR-amplified on a Bgl11-Xba1 fragment from pCherry10 (Carroll et al., 2010) and cloned into pG1A to make plasmid pCherry-Kan. A transcriptional fusion of the *rpmB2*-promoter with the mGreenLantern (mGL) fluorescent protein gene (Campbell et al., 2020) was synthesized (Thermo Fisher GeneArt) and subcloned into the Xba1 site of pCherry-Kan to make pRpmB2-mGL-Km-Alt (Figures 1A,B), which reports constitutive expression of mCherry and zinc responsive RpmB2-promoter-driven expression of mGreenLantern. The entire mCherry/mGreenLantern dual reporter region was also subcloned into pKINTA (Stewart et al., 2002) to make an chromosome-integrating construct: mCherry/mGreenLantern were PCR amplified with primers mGL-mCherry1 (5’ gcggagctcGTACCCGGGGATCTTCACTT 3′) and mGL-mCherry2 (5’ gcggagctcCTGCAGGTCGACTCTAGTGA 3′), cut with Sac1 and cloned into the Sac1 site of pKINTA to make pRpmB2-mGL-KINTA-Alt.

### 2.2 Bacterial strains and culture methods

*M. bovis* BCG Pasteur 1173P2 was maintained on Middlebrook 7H11 solid medium (Millipore) supplemented with 0.5% glycerol and 5% oleic acid dextrose catalase enrichment (OADC, ThermoScientific Remel). Liquid cultures were grown in Middlebrook 7H9 broth (Millipore) supplemented with 0.05% Tween-80 (Sigma-Aldrich), 0.2% glycerol and 10% albumin dextrose catalase enrichment (ADC, ThermoScientific Remel). Following transformation with pRpmB2-mGL-Km-Alt or pRpmB2-mGL-KINTA-Alt reporter constructs by electroporation, the plasmid was maintained in BCG by selection with 25 μg/mL kanamycin in solid and liquid culture medium. For some experiments, BCG was cultured in Sauton’s broth (Per Litre: KH_2_PO_4_ 0.5 g, citric acid 2 g, MgSO_4_.7H_2_O 0.5 g, ferric ammonium citrate 0.05 g, glycerol 23 mL, Tween-80200uL, L-asparagine 4 g). ZnSO4 was added as indicated. TPEN (N,N,N′,N′-tetrakis(2-pyridylmethyl)ethylenediamine) purchased from Sigma Aldrich was dissolved in DMSO at a concentration of 50 mM, and single use aliquots were stored at −20 °C.

### 2.3 Inhibition of BCG growth by zinc poisoning and zinc restriction

BCG bacilli were grown to mid log, adjusted to an optical density at 600 nm (OD600nm) of 0.05 in 7H9-ADC medium (which contains 6 μM zinc), and placed in a microtitre plates. Excess zinc sulphate and TPEN were added in 7H9-ADC at the concentrations shown, and the plates sealed and incubated for 7 days at 37 °C. The bacilli were resuspended using a multichannel pipette, and OD600nm read using a BioTek Epoch Microplate reader. The mean OD600nm value was calculated for each experiment from technical triplicate values in the microtitre plate. Normalised growth was calculated by dividing values by the mean of untreated wells from all experiments.

### 2.4 Zinc starvation with TPEN assays

Mid to late log phase cultures (OD600nm 0.7–1.0) of BCG-pRpmB2-mGL-Km-Alt grown in 7H9-ADC medium with 25 μg/mL kanamycin were diluted to 10% in fresh culture medium in universal tubes (6 mL per tube). TPEN was added to a final concentration of 5 μM, and a DMSO control was included. Bacilli were harvested and analysed at the stated timepoints.

### 2.5 Flow cytometry

0.3–0.5 mL of BCG was harvested from experiments at the indicated timepoints. The volume was adjusted to 1 mL with phosphate buffered saline (PBS), and centrifuged at 10,000 x *g* in a bench top centrifuge for 10 min. The bacilli were then resuspended in 1 mL PBS and analysed using an Attune NxT Flow Cytometer (ThermoScientific). Events were gated to exclude debris and doublets, and 10,000 events acquired. The mCherry-positive population was then selected, and the median fluorescent intentisy (MFI) of both mCherry and mGreenLantern for this population was recorded. The ratio of the values was calculated by dividing the MFI mGreenLantern value by the MFI mCherry value.

### 2.6 Confocal microscopy

Zinc-depleted and control cultures of BCG-pRpmB2-mGL-Km-Alt were loaded onto a CellASIC ONIX plate for bacteria (B04, Millipore), using a CellASIC microfluidic pump (Millipore). mCherry and mGreenLantern expression was analysed using a Nikon A1M Confocal Microscope, with a CFI Plan Apochromat Lambda D 40x air lens (numerical aperture 0.95), using NIS Elements software and ImageJ with FIJI plugins.

### 2.7 Extended stationary phase assays

BCG-pRpmB2-mGL-Km-Alt bacilli were expanded in 7H9-10% ADC 25 μg/mL kanamycin medium under shaking conditions, and analysed by flow cytometry to ensure basal levels of RpmB2-mGL expression. Thereafter, the bacilli were split into the indicated culture vessels and analysed at the indicated timepoints. For static cultures, replicate cultures were set up such that each sampling point remained static throughout the experiment until harvesting. OD600nm was measured in 1 mL cuvettes in a spectrophotometer (6,715 UV/Vis Spectrophotometer, Jenway). Bacteria were enumerated by serial dilution, plating on 7H11-5% OADC solid plates and counting of colony forming units (cfu).

### 2.8 Antibiotic tolerance assays

BCG-pRpmB2-mGL-Km-Alt under experimental conditions were placed in 1 mL aliquots in 2 mL sterile screw-capped tubes with 5 μg/mL streptomycin or 1 μg/mL rifampicin, and incubated at 37 °C for 7 days. Bacteria were enumerated on solid media by serial dilution on 7H11-5% OADC plates.

### 2.9 Statistical analysis

One-way ANOVA tests, two-tailed Student’s *t*-tests, and two-tailed *t*-tests with Welch’s correction were performed using GraphPad Prism software, as indicated in the figure legends. Cfu data were log10-transformed prior to statistical testing.

## 3 Results

### 3.1 Zinc restriction and zinc poisoning inhibit the growth of *Mycobacterium bovis* BCG

**Figure 2:**
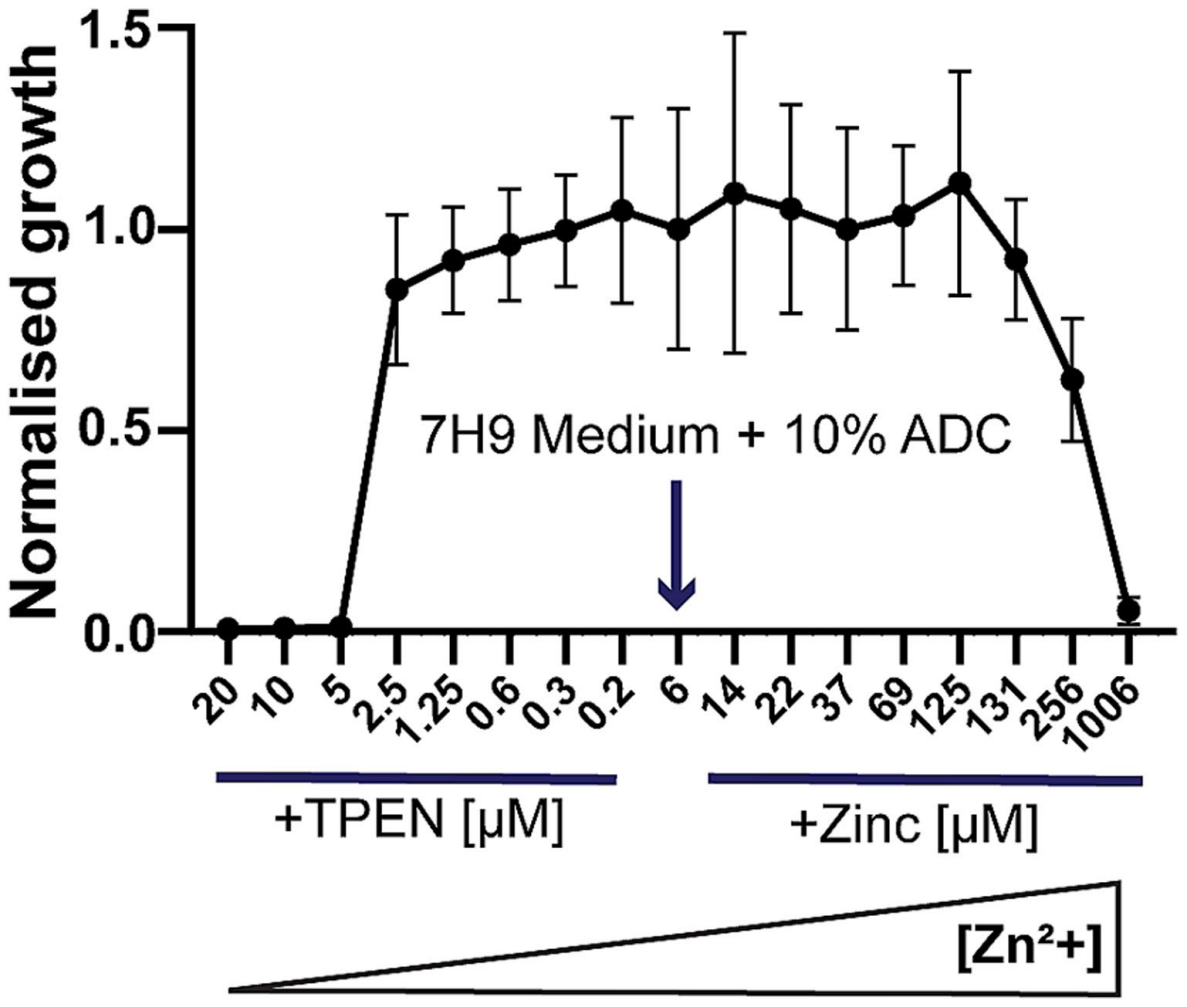
Zinc restriction and zinc poisoning inhibit the growth of *M. bovis* BCG. BCG bacilli were grown in 7H9-10% ADC medium, and seeded in microtitre plates with TPEN to chelate zinc, or with excess zinc. Growth (OD_600nm_) was assessed after 7 days. Results are mean of *N* = 3 independent experiments, +/− SD.

As a vital enzyme co-factor, adequate zinc provision is required for *M. tuberculosis* growth; however, in the caseum of the granuloma, zinc is chelated by calprotectin secreted by neutrophils. Zinc starvation leads to the upregulation of the Zur regulon, which bacilli use to counter the effects of zinc starvation. By contrast, excess zinc is pumped into the macrophage phagosome to poison the intracellular bacilli. To understand the range of effects of zinc starvation and zinc excess on *M. bovis* BCG, a model organism for *M. tuberculosis*, we cultured *M. bovis* BCG in microtitre plates in 7H9-10% ADC medium (which contains approximately 6 μM zinc) and added zinc to excess, or N,N,N′,N′- *tetrakis*-(2-Pyridylmethyl)ethylenediamine (TPEN) to chelate zinc. As seen in Figure 2, both zinc excess and zinc restriction inhibit the growth of *M. bovis* BCG.

### 3.2 Construction of an *rpmB2*-mGreenLantern fluorescent reporter strain of *Mycobacterium bovis* BCG

To assess the expression of the alternative ribosomal protein paralogue operon *rpmB2-rpmG1-rpsN2-rpsR2*, a reporter plasmid was constructed carrying the mGreenLantern (mGL) protein under the control of the *rpmB2* promoter, and the mCherry protein under the control of the constitutive sigma-70 G13 promoter from *M. marinum* (Barker et al., 1999) (Figure 1A). The *rpmB2* promoter region carries two Zur binding sites (Figure 1B). Under zinc replete conditions Zur repressor binding inhibits expression, whereas under zinc limiting conditions the operon is expressed. The expression of *rpmB2*-mGreenLantern in response to zinc concentration was tested by exposing the bacilli to zinc concentrations ordinarily experienced in standard 7H9-10% ADC medium (~6 μM zinc) and following chelation of zinc using TPEN. Expression was monitored by microscopy (Figure 1C) demonstrating an increase in *rpmb2*-mGreenLantern expression during zinc restriction. The bacilli were additionally monitored at the single cell level by flow cytometry, and the median fluorescent intensity (MFI) of rpmb2-mGreenLantern in the G13-mCherry-positive population was measured. As seen in Figure 1D, chelation of zinc with 5 μM TPEN induces *rpmB2*-mGreenLantern expression and this can be measured by flow cytometry. Studies using such dual-reporter systems in bulk microplate reader assays typically normalize the fluorescent signal from the inducible signal to the fluorescent signal from the constitutive promoter, and express this as a ratio. In addition to measuring the population rpmb2-mGreenLantern MFI, we also measured the population G13-mCherry MFI, and investigated these results both separately (Figure 1E) and as a ratio (Figure 1F) to mimic results that would be recorded in a bulk assay. As seen in Figure 1E, treatment with 5 μM TPEN caused a reduction in G13-mcherry MFI. Therefore, when these results are expressed as a ratio, the increase in ratio over time with 5 μM TPEN is in part due to the increase in rpmb2-mGreenLantern expression in response to zinc limitation, and also due to a decrease in G13-mCherry expression. Our results demonstrate the importance of measuring rpmb2-mGreenLantern on a per-cell basis by flow cytometry, rather than relying on normalization to constitutively expressed G13-mCherry. Therefore, throughout this study we have presented the population MFI of *rpmb2*-mGreenLantern. In addition, we have included the population MFI of G13-mCherry to allow a fuller understanding of the transcriptional state of the bacterium. We have also presented the ratio of these values to represent what would be measured by a bulk culture approach such as measurement by microplate reader to provide further context.

### 3.3 Zinc restriction does not lead to tolerance to streptomycin or rifampicin

**Figure 3:**
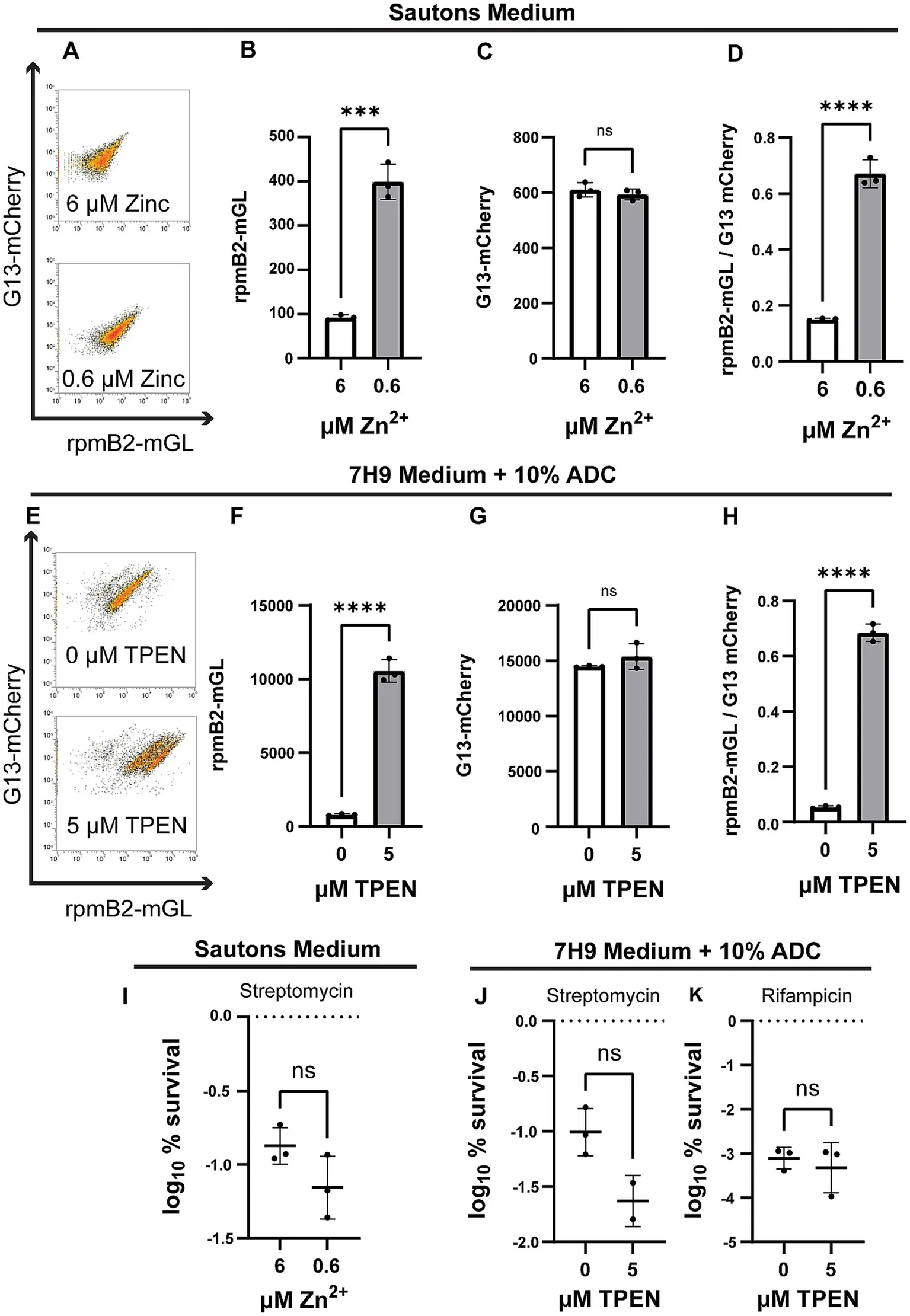
Antibiotic sensitivity of zinc-restricted BCG bacilli. BCG bacilli carrying the integrating ribosome reporter construct (BCG pRpmB2-mGL-KINTA-Alt) were cultured for 4 days in Sauton’s medium containing 6 μM zinc or 0.6 μM zinc **(A–D)** and analysed by flow cytometry. BCG bacilli carrying the alternative ribosome reporter construct (BCG pRpmB2-mGL-Km Alt) were cultured for 7 days in 7H9 medium + 10% ADC containing 0 μM or 5 μM TPEN to chelate zinc **(E–H)** and analysed by flow cytometry. The median fluorescent intensity of rpmB2-mGreenLantern (rpmB2-mGL) **(B,F)**, constitutively expressed G13-mCherry **(C,G)**, and the ratio of these values **(D,H)** is plotted. The bacilli were then exposed to 5 μg/mL streptomycin **(I,J)** or 1 μg/mL rifampicin **(K)** for a further 7 days. Data are mean +/− S.D. of *N* = 3 biological replicates (*N* = 2 biological replicates in 3J 5 μM TPEN treatment). Asterisks represent significant difference (*p < 0.05, **p < 0.01, ***p < 0.001, ****p < 0.0001, ns not significant) by two-tailed Students *t* test.

A study by Li et al. (2018, 2021) and Li Y. et al. (2020) demonstrated that zinc-starved *M. smegmatis* are phenotypically resistant to streptomycin, which is reported to occur due to the MPY protein binding to alternative ribosomes at the RNA-loading site, which occludes the binding sites of streptomycin and kanamycin. However, this finding was disputed by Tobiasson et al. (2019), and has not been demonstrated in *M. tuberculosis* complex organisms. To assess the role of zinc restriction and alternative ribosome expression in phenotypic resistance to streptomycin, BCG bacilli carrying the alternative ribosome reporter construct either as an episomal plasmid (pRpmB2-mGL-Km-Alt) or as a genomically integrated version (pRpmB2-mGL-KINTA-Alt) were subjected to zinc restriction by either culturing in Sauton’s medium with high (6 μM) or low (0.6 μM) concentrations of zinc (Figures 3A–D), or by culturing in 7H9-10% ADC medium with 5 μM TPEN for 7 days (Figures 3E–H). *rpmB2*-mGreenLantern reporter expression was measured by flow cytometry, and in parallel the bacilli were exposed to streptomycin for a further 7 days to assess survival. As seen in Figure 3, zinc restriction leads to induction of *rpmB2*-mGreenLantern expression in both Sauton’s medium (Figures 3A–D) and during TPEN treatment (Figures 3E–H). Zinc restriction did not change the sensitivity of the bacilli to streptomycin in either treatment (Figures 3I,J). Additionally, TPEN-treated bacilli were exposed to rifampicin for 7 days, and similarly showed no change in antibiotic sensitivity (Figure 3K). Thus, induction of the alternative ribosome by zinc depletion did not confer antibiotic resistance in *M. bovis* BCG.

### 3.4 Antibiotic tolerance during late stationary phase can occur with or without alternative ribosome expression

**Figure 4:**
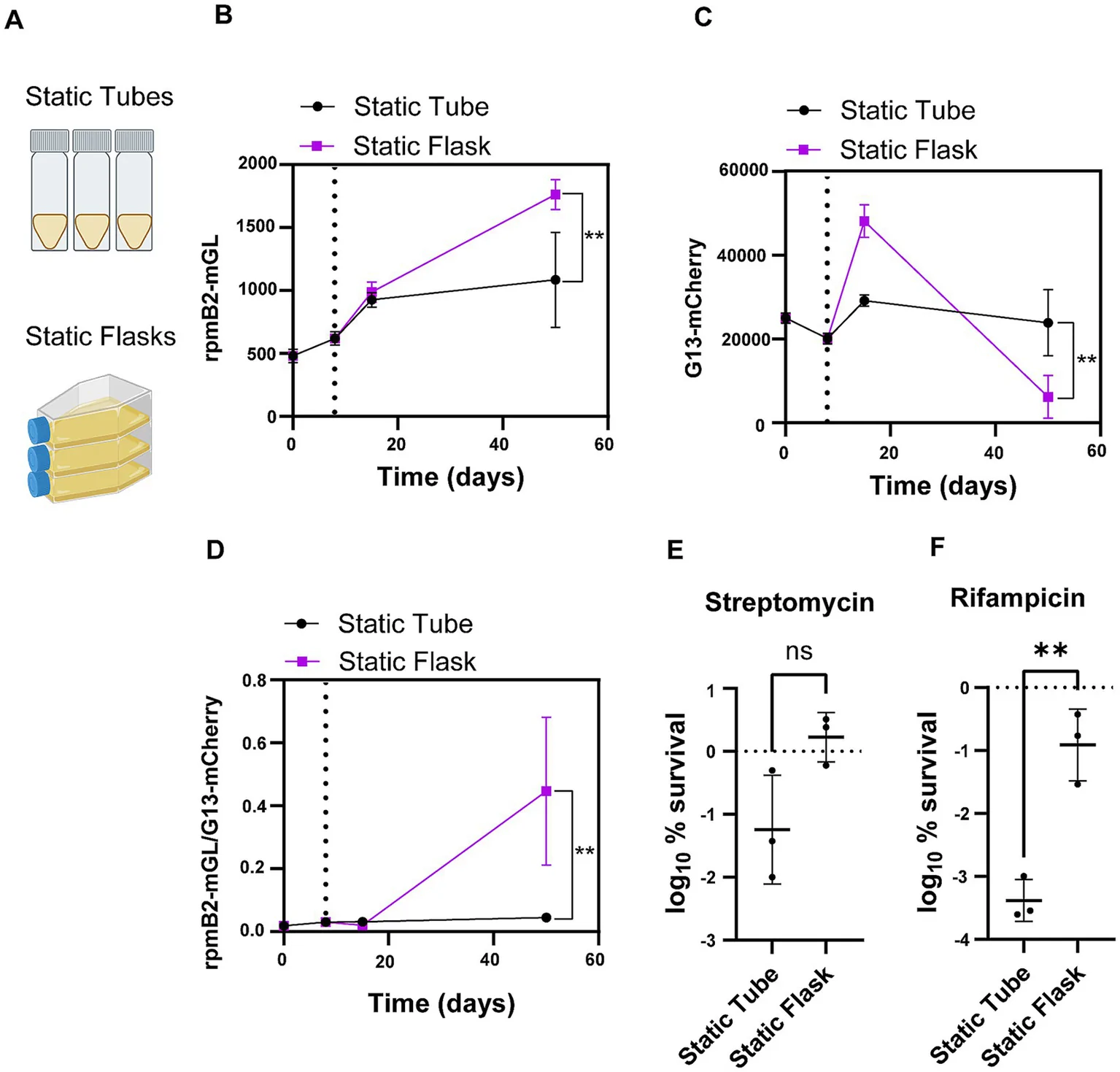
Expression of rpmB2-mGL in BCG during late stationary phase varies with culture conditions. BCG bacilli carrying the reporter construct were expanded in static tissue culture flasks, and at day 8 (dotted line) seeded into tubes or flasks **(A)** and cultured under static conditions. Bacilli were monitored by flow cytometry over 50 days **(B–D)**. The median fluorescent intensity of *rpmB2*-mGreenLantern (rpmB2-mGL) **(B)**, constitutively expressed G13-mCherry **(C)**, and the ratio of these values **(D)** is plotted. At 50 days, bacilli were treated with streptomycin **(E)** or rifampicin **(F)** for 7 days. Flow cytometry data **(B–D)** are mean +/− S.D. for *N* = 3 biological replicates at days 8 and 15, and *N* = 6 biological replicates at day 50. **(E,F)** data are mean +/− S.D. of 3 biological replicates. Asterisks represent significant difference (**p < 0.01, ns not significant) by two-tailed *t* test with Welch’s correction (BD), and by two-tailed Students *t* test **(E,F)**.

We next investigated if induction of the alternative ribosome during stationary phase was associated with antibiotic tolerance. The expression of *rpmB2*-mGreenLantern was assessed under zinc replete conditions (7H9 Medium + 10% ADC) during extended culture into late stationary phase. Preliminary observations found that stationary phase induction of *rpmB2*-mGreenLantern expression varied dependent on culture conditions such as vessel and agitation. We therefore compared antibiotic tolerance in *rpmB2-*mGreenLantern reporter BCG cultured in two contrasting conditions: universal tubes and tissue culture flasks. Bacilli were expanded in shaking flasks, and split into tubes and flasks that were cultured under static conditions (Figure 4A). Flow cytometry analysis at day 50 showed induction of *rpmB2*-mGreenLantern expression was much higher in bacteria from static flasks than from static tubes (Figures 4B,D). Additionally, these bacilli were more tolerant to rifampicin (Figures 4F), but were not significantly more tolerant to streptomycin although all the static flask cultures had greater survival in streptomycin than the low-*rpmB2* tube cultures (Figures 4E). This was consistent with the possibility that antibiotic tolerance in extended stationary phase could be caused or enhanced by elevated expression of the alternative ribosome proteins.

To help disentangle antibiotic tolerance caused by alternative ribosome expression from other physiological changes associated with extended stationary phase, we performed a second, longer comparison of BCG grown in these conditions. Over time, the cultures were analysed for expression of the alternative ribosome reporter fluorescence. Additionally, OD600nm and cfu/mL were measured to give a broader perspective of bacterial physiology. Finally, tolerance to 7-day treatment with streptomycin or rifampicin was measured at each sampling time.

**Figure 5:**
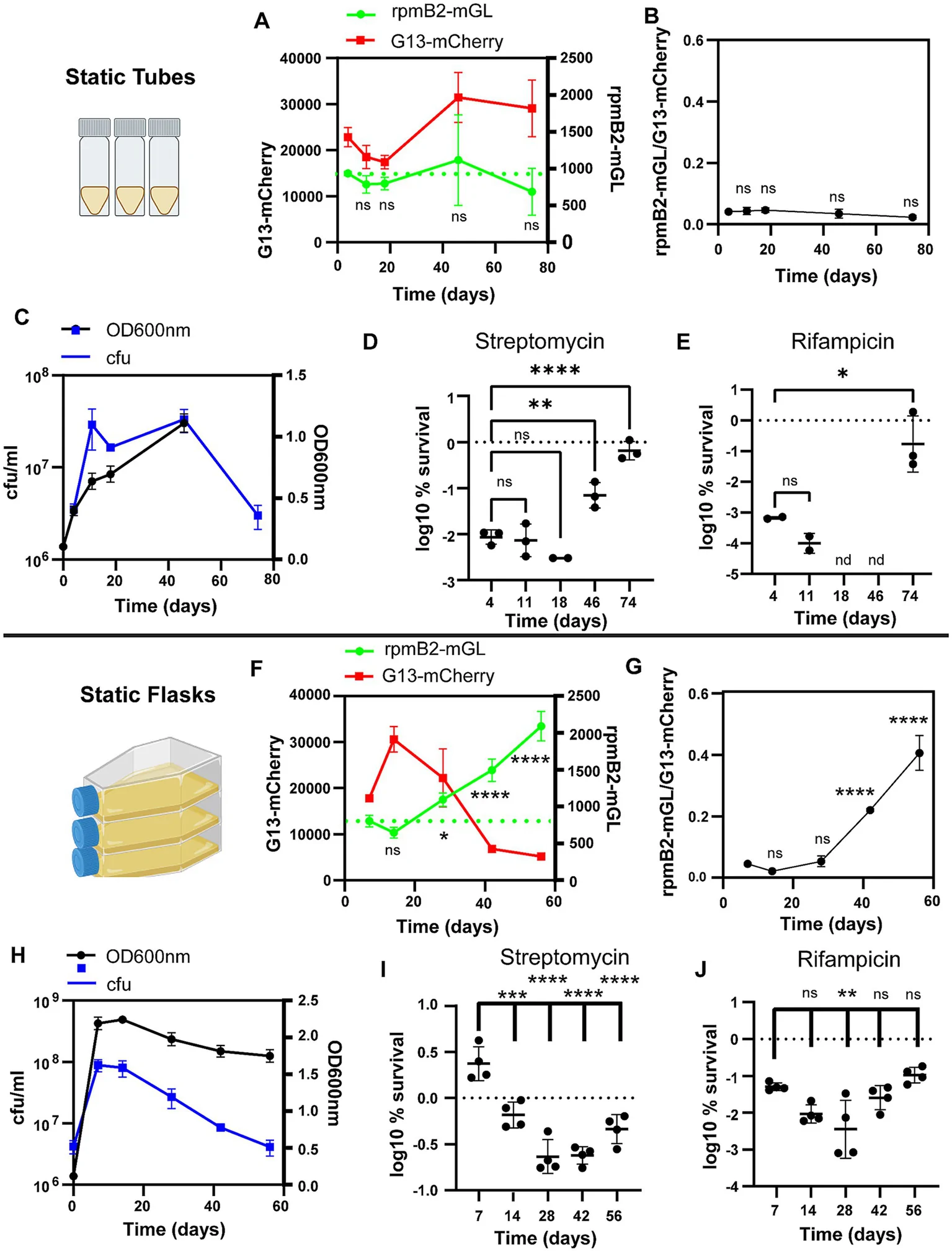
Emergence of antibiotic tolerance over time in stationary phase cultures of BCG-rpmB2-mGL alternative ribosome reporter bacilli. Bacilli were cultured in static tubes **(A–E)**, or static flasks **(F–J)**. At the indicated timepoints, bacilli were analysed by flow cytometry. The median fluorescent intensity of *rpmB2*-mGreenLantern (rpmB2-mGL), constitutively expressed G13-mCherry **(A,F)**, and the ratio of these values **(B,G)** is plotted, and OD600nm and cfu/mL were measured **(C,H)**. Antibiotic tolerance was assessed at the indicated timepoints by exposure to streptomycin **(D,I)** or rifampicin **(E,J)** for 7 days. Data are mean +/− SD of *N* = 3 **(A–E)** or *N* = 4 **(F–J)** biological replicates, except where data points show otherwise **(D,E)**. ND, not determined. Asterisks represent significant difference (*p < 0.05, **p < 0.01, ***p < 0.001, ****p < 0.0001, NS not significant) by One-way ordinary ANOVA with Dunnett’s multiple comparisons test **(A,B,D–G,I,J)**.

As seen in Figure 5, bacilli cultured in static tubes did not increase their levels of *rpmB2*-mGreenLantern expression during the time course (Figures 5A,B). By comparing OD600nm and cfu, (Figure 5C) it can be seen that the bacteria are entering a dormant growth phase at 46 days; at this point, the bacilli became more tolerant to streptomycin, which was further increased at day 74 (Figure 5D) when bacilli became resilient to rifampicin (Figure 5E) and lost culturability on solid media, as seen by a decrease in cfu detected (Figure 5C).

Bacilli cultured in static tissue culture flasks started to increase *rpmB2*-mGreenLantern expression at 42 days, which increased further at day 56 (Figure 5F). This was accompanied by a decrease in G13-mCherry expression (Figure 5F), leading to a sharp increase in the induction ratio of *rpmB2* (Figure 5G). Over time, the bacilli lost culturability on solid media (Figure 5H). The bacilli did not show increased tolerance to streptomycin or rifampicin over the time-course and in fact show decreased survival in streptomycin and rifampicin at day 28 (Figures 5I,J). There was an indication of increasing resilience thereafter to day 56 for both antibiotics which was concurrent with r*pmB2* induction.

Taken together, our data demonstrate that emergence of antibiotic tolerance in stationary phase can be concurrent with expression of alternative ribosomes (Figure 4), but alternative ribosome expression is not a pre-condition of antibiotic tolerance in stationary phase (Figure 5). Furthermore, cultures expressing high levels of alternative ribosomes under zinc restriction are not more tolerant to antibiotics, (Figure 3) and expression of alternative ribosomes during late stationary phase does not lead to tolerance in every case (Figure 5). Thus, our data suggest concurrent rather than causal development of antibiotic tolerance and expression of alternative ribosomes as the BCG enter late stationary phase.

## 4 Discussion

In this study, we used fluorescent mGreenLantern reporter to monitor alternative ribosome expression under conditions of zinc starvation and during stationary phase, and to correlate these findings with growth phase and antibiotic tolerance. We found that in the *M. tuberculosis*-complex organism *M. bovis* BCG, zinc starved bacilli, which have highly elevated expression of alternative ribosomes, are not more tolerant to streptomycin or rifampicin than zinc replete bacilli, a result in concordance with the work of Tobiasson on *M. smegmatis* (Tobiasson et al., 2019). We additionally monitored both the expression of alternative ribosomes and the emergence of antibiotic tolerance in extended stationary phase, and demonstrated that the expression of alternative ribosomes during extended stationary phase was highest during culture in static flasks. Furthermore, time course experiments demonstrated that while in some cases alternative ribosome expression was associated with increased tolerance to rifampicin, this was not a requirement because streptomycin and rifampicin tolerance developed over time irrespective of alternative ribosome expression. Taken together, this suggests antibiotic tolerance develops due to other changes in physiological state such as entering a slow growing, metabolically inactive phenotype rather than as a result of alternative ribosome expression *per se*.

In the present study, the alternative ribosome reporter construct contains both *rpmb2*-promoter driven mGreenLantern expression, and mCherry expression driven by the constitutively active G13 promoter. Due to our use of flow cytometry as a readout, we were able to gate for G13-mCherry positive events, and monitor the changes in expression of *rpmb2*-mGreenLantern in this population (expressed as MFI of the population). Through this primary analysis, we are able to analyse rpmB2-mGreenLantern expression without the need for normalization against the G13-mCherry signal. In addition, we measured the population MFI of G13-mCherry. Although considered a strong constitutive promoter (Carroll et al., 2010, 2014; Zelmer et al., 2012), we found that mCherry expression under the G13 promoter varied under different culture conditions. In this study we have presented the rpmB2-mGreenLantern MFI, the G13-mCherry MFI, and also the ratio of these two mean values. Our data demonstrate that when these values are expressed as a ratio, the change in G13-mCherry expression can distort the reported ratio. This would introduce significant bias in bulk microplate reader assays, where a reduction in G13-mCherry could artificially increases the rpmB2-mGreenLantern/G13-mCherry ratio the absence of increased rpmb2-mGreenLantern expression. This limitation could be overcome by designing and testing reporter constructs with less variably-expressed constitutive promoters, which could be adapted for use in microplate reader assays. Additionally, other gene-expression readouts such as RT-qPCR or RNA-seq could be used to confirm our results.

In our study, we have used the *rpmb2*-promoter region to drive mGreenLantern expression, however a further alternative (C-) ribosomal subunit RpmB1 (50S L28) is at a different genomic location (Rv0105c) under a different promoter and Zur box. The expression of *rpmB1* has been shown to be expressed under zinc starvation as part of the Zur-regulon along with *rpmB2* (Maciag et al., 2007), and transcription of both has also been demonstrated during stationary phase (Butler et al., 2025). The dependence of both alternative ribosome loci and of our reporter system on Zur de-repression during zinc starvation suggest that our reporter acts as a global indication of the response associated with the expression of alternative ribosomes during zinc starvation. However, further work to determine the expression of both promoters (for example by RNA-Seq) would help to experimentally evaluate this assumption and may provide insights into potential differences in expression of alternative ribosome paralogues.

Successful treatment of tuberculosis with drug therapy is hindered by the existence of persister populations of bacilli that are phenotypically tolerant to antibiotics. Alternative ribosome expression in *M. tuberculosis* is of considerable interest due to the association of the expression of the *rpmB2-rpmG1-rpsN2-rpsR2* operon in slow growing bacilli (Beste et al., 2007), antibiotic persisters (Keren et al., 2011), and in bacilli derived from zinc-starved sputum that is believed to be a source of persister bacilli (Garton et al., 2008; Lai et al., 2021). However, efforts to directly demonstrate antibiotic tolerance in these bacilli have yielded conflicting results. Experiments by Li et al. (2018, 2019, 2021) and Li Y. et al. (2020) demonstrated that tolerance to streptomycin could be induced in *M. smegmatis* cultures that were zinc starved, due to expression of “hibernating” alternative ribosomes. In their experiments, MRF (MSMEG_6069; Rv0106) recruits the ribosomal hibernation factor MPY (MSMEG_1878; Rv3241c) to the alternative ribosome which occludes the binding site of aminoglycoside antibiotics, and indeed of RNA itself, resulting in “hibernating” ribosomes that are transcriptionally inactive and tolerant to aminoglycoside antibiotics. However, the control bacilli used were cultured in 1 mM zinc, a concentration that is supra-physiological (Chen et al., 2020), and indeed may be toxic. Under mild zinc deprivation, where bacilli are not in complete stasis, alternative ribosomes were found to be transcriptionally active, but with a different transcriptional profile (Chen et al., 2020). Li et al. (2018) also found that MPY is expressed constitutively, and recruitment to alternative ribosomes requires the action of MRF. MRF itself is subject to two levels of zinc regulation; firstly, it is part of the zur-regulon (Supplementary Table 1) and expression is induced by zinc deprivation. Secondly, it is degraded by proteases in the presence of zinc. Together this provides a mechanism for alternative ribosome hibernation exclusively under extreme zinc deprivation. However, work by other researchers has found that MPY is associated with primary zinc-dependent ribosomes during stationary phase in *M. smegmatis* (Mishra et al., 2018) and during stationary phase stasis during hypoxia or carbon limitation in *M. smegmatis* (Trauner et al., 2012). MPY expression itself is induced during carbon limitation and slow growth in *M. smegmatis* (Berney and Cook, 2010), and during PBS starvation in *M. tuberculosis* (Betts et al., 2002; Sawyer et al., 2021; Sawyer and Cortes, 2022). The interaction of MPY with alternative ribosomes was not determined in our current study, and it would be of considerable interest to assess the composition of stationary phase ribosomes and their hibernation factors. The co-occurrence of tolerance to streptomycin and rifampicin in some of our experiments suggests that the overall metabolic and replicative status of the bacteria is an important factor. Although calprotectin protein secreted by neutrophils is known to sequester Zn2+, levels of free zinc in sputum samples are difficult to measure and may vary greatly (Marcantonio et al., 2025b) and it is unclear whether zinc concentration is reduced enough to trigger MRF/MPY-dependent alternative ribosome hibernation *in vivo*. Restriction of other key nutrients *in vivo* may additionally influence ribosome hibernation. Finally, there may be differences in the regulation of alternative ribosomes and their hibernation factors between *M. tuberculosis*-complex organisms and *M. smegmatis*. Taken together, our data caution against presuming antibiotic tolerance occurs when bacilli are found to express alternative ribosomes.

The increased expression of alternative ribosomes in slow growth and stationary phase without zinc starvation has been observed by multiple research groups, and suggests that zinc starvation is not the only mechanism by which they are regulated. Indeed, we have previously shown that optimal expression of the zur regulon requires ADP-riboslyation of the zur-box to stabilise the off-state of the repressor (Butler et al., 2025). CmtR has been shown to inhibit zur binding during oxidative stress, allowing the expression of ESX-3 in *M. bovis* BCG and zinc uptake to counter oxidative stress (Li X. et al., 2020). Depletion of *M. tuberculosis* protein kinase B (PknB) leads to alternative ribosome expression by reduced phosphorylation of the repressor Lsr2 (Alqaseer et al., 2019). Together these results suggest complex regulation of alternative ribosome expression that does not rely solely on zinc starvation, which again may contribute to the observations that sometimes alternative ribosome expression correlates with tolerance and sometimes it does not.

Our study is not without limitations. While we have calculated the zinc concentrations that have been added to our assays, we have not directly measured these values. As zinc is a common contaminant of laboratory glassware and solutions (Kay, 2004), the concentration of zinc in our assays is likely to be higher than reported. We have used TPEN to restrict zinc in our assays, and TPEN is able to chelate other metal ions which may also contribute to bacterial growth inhibition. However, other researchers have observed that the growth inhibitory effects of TPEN on *M. tuberculosis* can be relieved by introducing excess zinc, suggesting that zinc chelation is responsible for growth inhibition in our assays (Nelson et al., 2023). We did not determine the differences between static culture in tubes or tissue culture flasks that are responsible for increased rpmB2-mGL expression in tissue culture flasks. It is possible that extended culture in tissue culture flasks creates a micro-anaerobic growth environment or mimics the effects seen in biofilm growth, both of which are also associated with drug tolerance (Wayne and Sohaskey, 2001; Ojha et al., 2008). Additionally, further work would also be required to assess the contribution of the flask coating to this phenotype, and whether zinc starvation plays a role or if other regulatory mechanisms are responsible.

The extremes of zinc concentration experienced by *M. tuberculosis* within the host, from caseum to phagosome, lead to a series of varying microenvironments within the host that are a potential source of phenotypic heterogeneity in the bacteria themselves. Although our data do not support a direct effect of zinc starvation on the development of persisters through alternative ribosome expression, the differences in growth rate due to varying concentrations of zinc may form an important part of persister formation. Indeed, alternative ribosomes may be important for bacterial survival during stationary phase and in low zinc conditions, which may be important for the longevity of persister populations and for persistent infection. Additionally, other forms of phenotypic heterogeneity caused by varying zinc concentrations may be important to the outcome of infection, such as changes in translational ability of alternative ribosomes (Chen et al., 2020; Shen et al., 2025). Furthermore, zinc-starved *M. tuberculosis* bacilli are more resistant to oxidative stress (Dow et al., 2021), are phagocytosed more than their zinc-replete counterparts by macrophages, stimulate higher levels of ROS production, higher levels of macrophage death, and a more robust proinflammatory cytokine response (Marcantonio et al., 2025a). That zinc-starved *M. tuberculosis* are found in the sputum and are likely representative of the population responsible for transmission remains of considerable interest and importance to the field.

## Data Availability

The raw data supporting the conclusions of this article will be made available by the authors, without undue reservation.
